# Th1 Biased Progressive Autoimmunity in Aged *Aire-*Deficient Mice Accelerated Thymic Epithelial Cell Senescence

**DOI:** 10.14336/AD.2018.0608

**Published:** 2019-06-01

**Authors:** Jie Zhang, Yuqing Wang, Abudureyimujiang Aili, Xiuyuan Sun, Xuewen Pang, Qing Ge, Yu Zhang, Rong Jin

**Affiliations:** Department of Immunology, School of Basic Medical Sciences, Peking University Health Science Center, Key Laboratory of Medical Immunology, Ministry of Health (Peking University), Beijing 100191, China

**Keywords:** Aire, thymic senescence, thymic epithelial cells, autoimmune disease, Th1

## Abstract

Although autoimmune diseases, such as rheumatoid arthritis and systemic lupus erythematosus, are frequently associated with premature aging of the thymus, a direct link is missing between autoimmunity and thymic atrophy. Here we monitored the progression of thymic involution in *Aire-*deficient mice, in which defective negative selection causes spontaneous and progressive development of autoimmunity. In young and middle-aged mice, *Aire* deficiency appeared to be protective as supported by the reduced β-gal^+^ epithelial cells and the enhanced thymic output. However, once the autoimmune phenotype was fully developed in aged *Aire-*deficient mice, their thymuses underwent accelerated involution. In comparison to the age-matched wildtype littermates, old *Aire-*deficient mice showed lower numbers of total thymocytes and recent thymic emigrants but more β-gal^+^ thymic epithelial cells. This phenomenon may partly be attributable to the increased number of activated Th1 cells homing to the thymus. This speculation was further supported by the enhanced thymic aging following repeated challenges with complete Freund’s adjuvant immunization. Taken together, the present study highlights a unique mechanism by which autoimmunity facilitates the senescence of thymic epithelial cells through returning Th1 cells.

Thymic involution is characterized by reduced tissue mass and cellularity, disorganized morphology, and diminished generation and exportation of naïve T cells [1]. Loss of thymic function and T cell receptor (TCR) diversity is thought to contribute to impaired immunosurveillance. Thus, the decline of thymic function in aged individuals is linked to increased morbidity and mortality in a wide range of clinical settings such as opportunistic infections, autoimmunity and cancer [1, 2]. Better understanding of the mechanisms underlying thymic involution under physiological and pathological conditions is important for the development of strategies for medical intervention of immunosenescence-associated health problems in an aging world.

Several hypotheses have been proposed to explain thymic atrophy, including aging hematopoietic progenitors, dysfunction of thymic stroma and systemic factors such as elevating levels of sexual hormones [3]. During the acute infection, bulk of studies showed that pro-inflammatory factors induced acute but transient thymic atrophy characterized by loss of double positive (DP) thymocytes [4]. In contrast, the age-associated thymic atrophy primarily affects the epithelial compartment, leading to reduced thymic medullary islet complexity and impaired expression of tissue-restricted antigens [5-10]. Deteriorating epithelial compartment perturbs negative selection with increased release of autoreactive T cells [11]. Moreover, impaired T-cell development and decreased thymic output induces homeostatic proliferation of the peripheral T cell pool, with preferential expansion of autoreactive T cells along with an excessive production of IFN-γ. As such, aging is an important risk factor for the development of autoimmune diseases [12-14]. On the other hand, there is evidence that autoimmunity facilitates thymic involution. Premature aging of the thymus, for example, has been reported in young patients with autoimmune diseases such as rheumatoid arthritis (RA) and systemic lupus erythematosus (SLE) [14-16]. However, it remains elusive what links these two events.

Naïve CD4^+^ T helper (Th) cells have the potential to develop into multiple subsets of effector cells, such as Th1, Th2 and Th17 [17]. They are not only essential for orchestrating host defense against invading pathogens, but also contribute to the pathogenesis of autoimmunity. The important role of IFN-γ-producing Th1 cells have been demonstrated in multiple autoimmune disorders, both in human and in mouse. Patients with SLE, Sjögren’s syndrome and systemic sclerosis were showed to have increased mRNA levels of IFN-γ and T-bet in peripheral blood mononuclear cells (PBMC) [18]. In SLE patients with diffuse proliferative nephritis, elevated serum IFN-γ levels and IFN-γ/IL-4 (Th1/Th2) ratio was found to be correlated with disease activity [19]. Treatment with anti-IFN-γ antibody and soluble IFN-γ receptor was able to reduce serum levels of IFN-γ and disease severity in MRL-Fas^lpr^ and NZB/W F1 mice [18]. In contrast, local administration of IFN-γ or tissue-specific transgene-mediated expression of IFN-γ at inflammatory sites exacerbated disease in arthritis and autoimmune diabetes models [20]. And deletion of *Ifng* ameliorated nephritis in the MRL/lpr model of SLE [21]. Collagen-induced arthritis (CIA) and experimental autoimmune encephalomyelitis (EAE), on the other hand, seem to be predominantly mediated by Th17 cells [22]. In fact, *Ifng* or *Ifngr* deficiency accelerated onset and increased incidence of CIA and EAE, indicating a protective role of IFN-γ in suppressing the Th17 responses [20]. Given the potential link between autoimmunity and thymic involution, it would be interesting to see whether the various Th subsets induced by autoimmunity have a feedback role in the modulation of thymic functions.

Autoimmune regulator (Aire) is critically involved in the induction of central tolerance by driving the ectopic expression of a wide range of tissue-restricted antigens in medullary thymic epithelial cells (mTECs) [23-25]. Its mutation results in the development of autoimmune polyglandular syndrome type 1 (APS1), a rare and complex autoimmune disease in human [25]. *Aire-*deficient mice are similarly predisposed to autoimmunity. But the murine disorder is generally milder with a delayed onset [26]. Therefore, *Aire-*deficient mice provide a good model for studying the impact of autoimmunity on the thymic involution. In the present study, we compared thymic involution in *Aire-*deficient mice and the wildtype (WT) littermates. Differential impacts were observed on mice of different ages. While early thymic degeneration was displayed in the absence of *Aire*, this process was accelerated in aged *Aire*-deficient mice, possibly due to the increased recirculation of activated CD4^+^ T cells into the thymus.

## MATERIALS AND METHODS

### Mice

C57BL/6 congenic mice were purchased from Peking University Health Science Center (Beijing, China). B6.129S2-*Aire^tm1.1Doi^*/J (*Aire^+/-^*) and FVB-Tg (Rag2-EGFP)1Mnz/J mice were purchased from Jackson Laboratory (Bar Harbor, ME). FVB-Tg (Rag2-EGFP) 1Mnz/J mice were backcrossed for 10 generations onto the C57BL/6 background (termed as RAG2p-GFP in this paper). *Aire*^-/-^ mice were bred with RAG2p-GFP to generate *Aire*^-/-^ RAG-GFP mice. The animals were kept in a specific pathogen-free facility at Peking University Health Science Center (Beijing, China). The experimental procedures on use and care of animals had been approved by the ethics committee of Peking University Health Science Center No.LA2014178.

### Reagents

Anti-mouse CD4 PE-Cy7 (RM4-5), anti-mouse CD8a PE (53-6.7), anti-mouse CD62L FITC (MEL-14), anti-mouse Ly51 PE (BP-1) were purchased from BD Biosciences (San Diego, CA). Anti-mouse CD25 PE (PC61.5), anti-mouse CD44 APC (IM7), anti-mouse CD69 PerCP-Cy5.5 (H1.2F3), anti-mouse CD45 PerCP (30-F11), anti-mouse MHC-II PE (M5/114.15.2), anti-mouse IL-2 FITC (JES6-5H4), anti-mouse IFN-γ APC (XMG1.2), anti-mouse IL-4 FITC (BVD6-24G2), anti-mouse IL-17A PE (eBio17B7) and anti-mouse Foxp3 APC (3G3) were obtained from eBioscience (Waltham, MA). Anti-mouse EpCam PE-Cy7 (G8.8) was purchased from Biolegend (San Diego, CA).

### Flow cytometry

For recent thymic emigrant (RTE) analysis, the GFP fluorescence intensity was used to determine GFP^+^ (RTEs) and GFP^-^ (non-RTEs) cells in *Aire^-/-^* RAG2p-GFP transgenic mice. For detection of surface molecules, T cells were labeled with the appropriate fluorescent mAbs on ice for 30 min. For detection of cytoplasmic molecules Foxp3, T cells were collected, stained with surface molecules, fixed and permeabilized with the Foxp3/Transcription Factor Staining Buffer (eBioscience), and stained with APC-Foxp3 antibody on ice for 30 min. Flow cytometry was then conducted on a Beckman FACS Galios (Beckman Coulter) and data analysis was performed using Kaluza software.

### Intracellular cytokine staining

CytoSpot assay was performed with the Cytofix/ Cytoperm and GolgiStop kit according to the manufacturer’s instructions (BD Biosciences). Sorted GFP^-^CD4^+^ thymocytes, peripheral GFP^+^CD4^+^ T cells and GFP^-^CD4^+^ T cells were stimulated with plate-bound anti-CD3 and anti-CD28 for 12 hours. GolgiStop (brefeldin A 10μg/ml) was added to the culture in the last 4 hours. Cells were fixed by IC fixation buffer for 20 min at room temperature and permeabilized by incubating with Permeabilization Buffer for 10 min at room temperature. After washing, cells were collected and stained with fluorochrome-labeled anti-IL-2, anti-IFN-γ, anti-IL-4 and anti-IL-17A antibodies (1:400 dilution) for 30min on ice. Flow cytometry was then conducted on a Beckman FACS Galios (Beckman Coulter) and data analysis was performed using Kaluza software.

### In Vivo BrdU Labeling

For BrdU administration, mice received two intraperitoneal injections of BrdU (1 mg each at 4h intervals). Lymph nodes were harvested 1 h after the second injection. BrdU incorporation was detected using the APC BrdU Flow Kit (BD Biosciences). After surface staining, cells were fixed and permeabilized with Cytofix/Cytoperm buffer for 30 min on ice and then treated with DNase to expose incorporated BrdU. Subsequently, cells were stained with APC-conjugated anti-BrdU antibody.

### Senescence-associated β-galactosidase assay

Cryosections of thymus tissues of different age (16 μm thick) were analyzed for β-gal activity using a Senescence β-Galactosidase Staining Kit according to the manufacturer’s protocol (Cell Signaling Technology, Inc., Danvers, MA, USA, no.9860). Cryosections of thymus from CFA group were stained with anti-mouse K5 antibody followed by Cy5-conjugated goat anti-rabbit antibody (Abcam). Images were acquired with Olympus IX51. For quantification analysis, 10 randomly chosen sections were counted using Image J.

### Histology

Organs from *Aire^-/-^* mice and WT littermates at indicated age were collected and fixed overnight in 10% formalin, embedded in paraffin, sectioned and stained with hematoxylin and eosin (H&E). The degree of lymphocytic infiltrates was evaluated in a blinded fashion. Images were acquired with Olympus IX51. The brightness and contrast were adjusted in Adobe Photoshop CS.

### Immunization protocol

Mice were immunized subcutaneously in the flanks of the lower back, with 5μg of chicken ovalbumin (OVA grade V; Sigma-Aldrich) in 100μl saline or 100μl of complete Freund’s adjuvant (CFA, Sigma-Aldrich) for a total 6 times at 6-, 8-, 10-, 17-, 19- and 21-week-old. At 16-, 27- and 39-week-old, blood, thymus, spleen and the peripheral lymph nodes were collected for phenotype analysis. Serum samples were stored at -80 °C.

### Isolation of thymic epithelial cells (TECs)

The thymus was removed and cut into small pieces (< 1 mm^3^) and digested with 2 mg/ml collagenase type IV (Gibco) and 0.1 mg/ml DNase I (Sigma) at 37 °C for 30 min with vortexing every 5 min. Cells from the last 3 digestions were collected. After depletion of thymocytes using anti-CD45 immuno-magnetic bead (Miltenyi Biotec), cells were stained with anti-mouse CD45 PerCP and anti-mouse EpCam PE-Cy7 on ice for 30min. After washing, TECs (CD45^-^EpCam^+^) were sorted on a FACS AriaⅡ (BD Biosciences). The purity of the sorted population was > 98%.

### Quantitative PCR

RNA was purified from TEC using Trizol reagent (Invitrogen). cDNA was synthesized using reverse transcription kit (Promega). Quantitative PCR was performed with primers as follows: *Il6* (5’-CTGCAAGAGACTTCCATCCAG-3’ and 5’-AGTGGT ATAGACAGGTCTGTTGG-3’); *Lif* (5’-ATTGTGCC CTTACTGCTGCTG-3’ and 5’-GCCAGTTGATTCTTG ATCTGGT-3’); *Osm* (5’-CCCGGCACAATATCCTC GG-3’ and 5’-TCTGGTGTTGTAGTGGACCGT-3’); *Ifng* (5’-TCAAGTGGCATAGATGTGGAAGAA-3’ and 5’-TGGCTCTGCAGGATTTTCATG-3’); *Il4* (5’-ACAGGAGAAGGGACGCCAT-3’ and 5’-GAAGCCCTACA GACGAGCTCA-3’); *Actb* (5’-TATGGAATCCTGTG GCATC-3’ and 5’-GTGTTGGCATAGAGGTCTT-3’). The quantification was based on delta delta CT calculations and was normalized to β-actin as loading controls.

### Statistics

Data are presented as mean values ± standard deviation (SD). Statistical significance between two groups was evaluated by two-tailed unpaired Student *t* test. Throughout the text, figures and figure legends, the following terminology is used to denote statistical significance: *p<0.05, **p<0.01, ***p<0.001.

**Figure 1.**
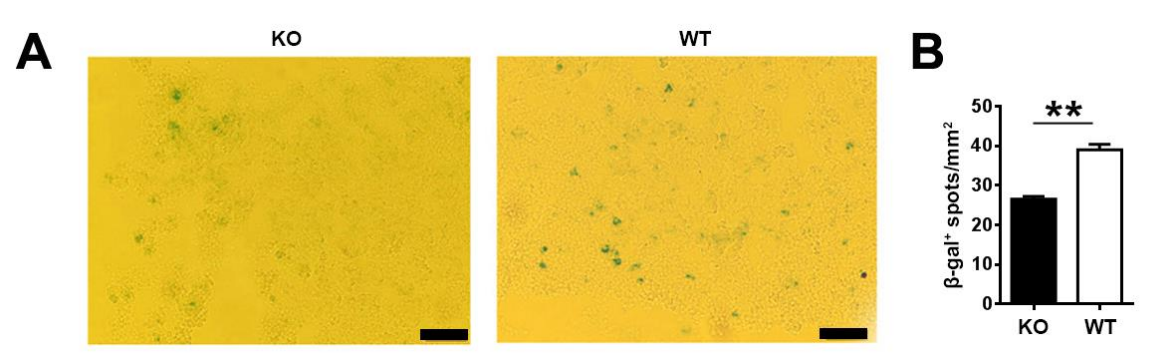
Decreased senescent cells in young *Aire*-deficient mice (**A**) Senescence-associated β-galactosidase (β-gal) was stained in thymic frozen slices from 6-week-old mice. Scale bars: 50μm. Representative images from three independent experiments are shown. (**B**) The number of β-gal^+^ senescent cells (blue) in 10 randomly chosen sections were counted using Image J. The statistical data are shown as average β-gal^+^ spots/mm^2^±SD (n=3 pairs). Statistical differences between groups were determined by the Student’s *t* test. **p < 0.01.

## RESULTS

### Aire deficiency was associated with delayed thymic involution in young and middle-aged mice

Thymic epithelial cell senescence was one of the major changes in age-related thymic involution. Aire is differentially expressed in medullary thymic epithelial cell (mTEC) subsets, absent in immature MHC-II^lo^CD80^lo^ mTECs, high in mature MHC-II^hi^CD80^hi^ mTECs, and low in the terminally differentiated MHC-II^lo^CD80^lo^involucrin^+^ mTECs [27]. Apart from its best-known function in the induction of central tolerance, *Aire* deficiency results in reduced terminally differentiated involucrin^+^ mTECs [28]. To investigate a potential role of Aire in the senescence of thymic epithelial cells (TECs), we first examined thymic slices from *Aire*^-/-^ mice on C57BL/6 background at the age of 6 weeks, a time point before the onset of autoimmune disease [26]. Senescent cells are metabolically active featured by increased β-galactosidase (β-gal) activity [29]. We found fewer β-gal^+^ senescent cells in thymic frozen slices from *Aire-*deficient mice. These β-gal^+^ cells morphologically represented epithelial cells (Fig. 1A and B).

**Figure 2.**
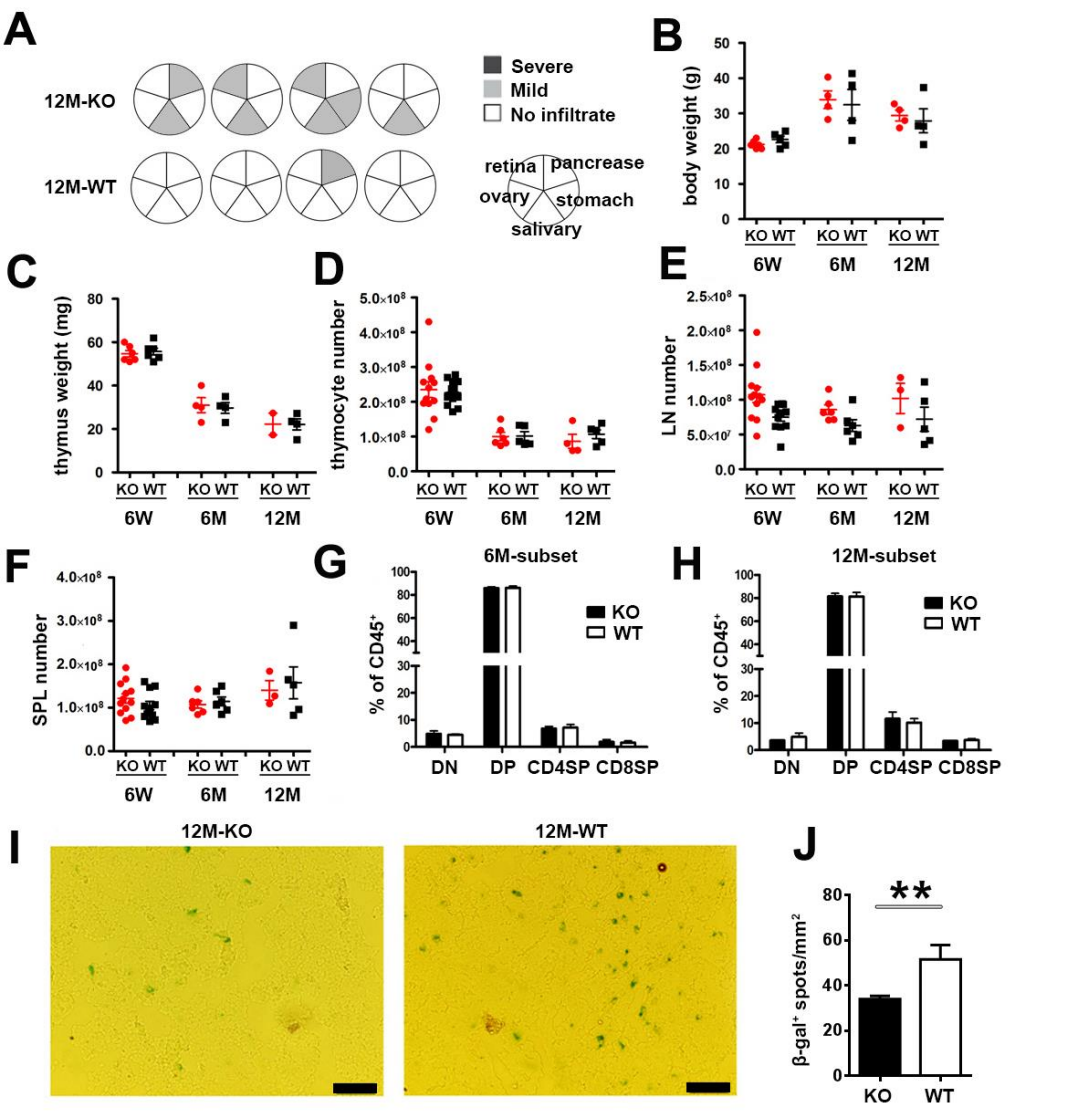
The autoimmune disorders in early middle-aged *Aire*-deficient mice (**A**) Lymphocyte infiltration in the salivary gland, retina, pancreas, stomach and ovary in 12-month-old WT (n=4) and *Aire*^-/-^ (n=4) mice. The circles represent individual mice and summarize the degree and pattern of immune infiltration in the indicated organs. Mouse body weight (**B**), thymus weight (**C**), the cell number of thymocytes (**D**), lymph node cells (**E**) and splenocytes (**F**) of 6-week-old, 6- and 12-month old *Aire*^-/-^ mice and WT littermates are shown. Each dot represents an individual mouse. (G, H) The percentage of double negative (DN), double positive (DP), CD4 and CD8 single positive (SP) thymocytes in 6-(**G**) and 12-month-old (**H**) mice were gated on CD45^+^ thymocytes. The statistical data are shown as Mean ± SD (n=8 pairs). No significant difference was detected in the above figures. (**I, J**) β-gal was stained in thymic frozen slices from 12-month-old mice (**I**). Scale bars: 50μm. (**J**) The number of β-gal^+^ senescent cells (blue) in 10 randomly chosen sections were counted using Image J. The statistical data are shown as average β-gal^+^ spots/mm^2^ ± SD (n=3 pairs). The experiments were repeated 3 times. Statistical differences between groups were determined by the Student’s *t* test. **p < 0.01.

To explore the impact of autoimmune disorders induced by *Aire* deficiency on the thymic involution, we examined the general aspects of the thymic function during mouse ontogeny. Although the autoimmune symptoms were mild on C57BL/6 background, lymphocyte infiltration was detected in the salivary gland in all 12-month-old *Aire*^-/-^ mice (Fig. 2A), indicating the onset of autoimmunity at that time point. However, the body weight, thymus weight and the number of thymocytes, lymph node cells and splenocytes were comparable between *Aire*^-/-^ mice and WT littermates before 12 months of age (Fig. 2B-F). Further analysis showed that the proportions of major thymocyte subsets were also similar (Fig. 2G and H). The number of β-gal^+^ senescent cells in thymic frozen slices was still significantly lower in *Aire-*deficient mice than in WT mice at the age of 12 months (Fig. 2I and J).

**Figure 3.**
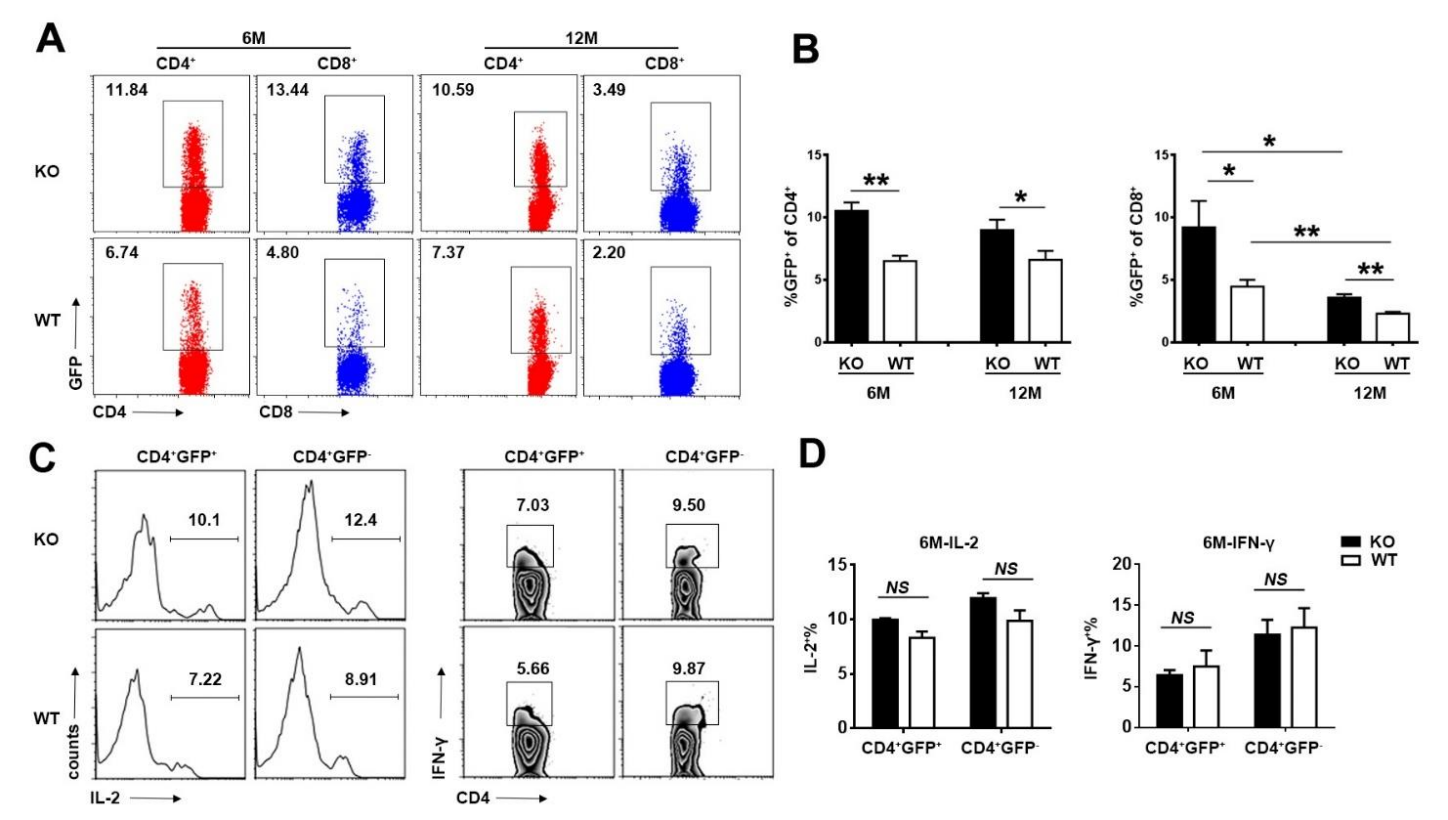
The alteration of recent thymic emigrants (RTEs) in early middle-aged *Aire*-deficient mice (**A, B**) The proportion of GFP^+^ cells in peripheral CD4^+^ and CD8^+^ cells in 6- and 12-month-old *Aire^-/-^* mice and WT littermates. Representative dot plots (**A**) and the statistical data (**B**) are shown. (**C, D**) Splenocytes from 6-month-old *Aire^-/-^* and WT littermates were stimulated with anti-CD3 and anti-CD28 for 6h. IL-2 and IFN-γ producing cells in the GFP^+^CD4^+^ and GFP^-^CD4^+^ T cells was determined by intracellular staining. Representative plots (**C**) and the percentage of IL-2^+^ and IFN-γ^+^ cells (**D**) are shown. Statistical data are presented as Mean ± SD (n=8 pairs). The experiments were repeated four times. Statistical differences between groups were determined by the Student’s *t* test. *p< 0.05, **p < 0.01, and *NS*, no significance.

Reduced thymic egress is a major character of thymic degeneration. To analyze the recent thymic emigrant (RTE) compartment in *Aire-*deficient mice at different ages, we crossed the *Aire^-/-^* mice with Rag2p-GFP mice, in which RTEs in the periphery are readily distinguished for their GFP expression [30]. The proportion of GFP^+^ cells in peripheral CD4^+^ and CD8^+^ cells was higher in 6-month-old *Aire*^-/-^ mice than in WT littermates, which was maintained until 12 months of age. The ratio of GFP^+^CD4^+^ cells was stable from 6-month- to 12-month-old mice, while the ratio of GFP^+^CD8^+^ cells was significantly decreased during this period both in *Aire*^-/-^ and WT mice (Fig. 3A and B). The pattern of the cell numbers is consistent with the percentage (data not shown). Concomitantly, the proportion of IL-2 and IFN-γ producing cells in the GFP^+^CD4^+^ and GFP^-^CD4^+^ T cells from *Aire*^-/-^ mice was comparable to WT mice, indicating the function of peripheral T cells was not altered (Fig. 3C and D). Together with the result of β-gal staining, the enhanced thymic egress indicate that *Aire* deficiency delayed thymic involution in young and middle-aged mice.

**Figure 4.**
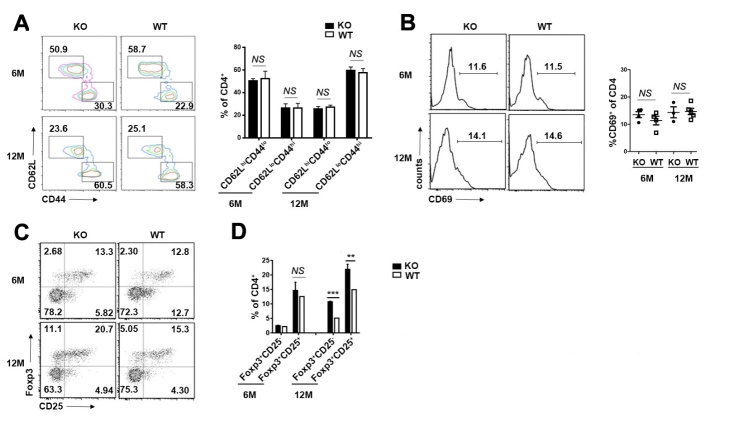
The activation of peripheral CD4^+^ T cells and the accumulation of Tregs in early middle-aged *Aire^-/-^* mice (**A**) CD62L and CD44 staining for splenic CD4^+^ T cells in 6- and 12- month-old mice. The number indicates the percentages of CD4^+^CD62L^high^CD44^low^ (naïve), CD4^+^CD62L^low^CD44^high^ (effector/memory) among CD4^+^ T cells (*left*). The statistical data are shown on the right as Mean ± SD (n=8 pairs). (**B**) CD69 staining for splenic CD4^+^ T cells in 6- and 12-month-old mice (*left*). The percentage of CD69^+^ T cells in CD4^+^ T cell are presented as Mean ± SD (*right*). Each dot represents an individual mouse. (**C**) Flow cytometric analysis of Tregs in the splenocytes of 6- and 12-month-old mice. Dot plots show representative profiles for CD25/Foxp3 staining (gated on CD4^+^ cells). (**D**) The percentage of Foxp3^+^CD25^-^ and Foxp3^+^CD25^+^ Tregs are shown as Mean ± SD (n=8 pairs). The experiments were repeated 3 times. Statistical differences between groups were determined by the Student’s *t* test. **p< 0.01, ***p < 0.001, and *NS*, no significance.

The delayed thymic output disrupted peripheral T cells homeostasis in 3-week-old *Aire^-/-^* mice [31]. To further explore the alteration of peripheral T cells in early middle-aged *Aire*^-/-^ mice, we compared their activation in the two groups. There was a similar frequency of activated/memory (CD62L^lo^CD44^hi^) T cells, as well as the expression of activation marker CD69 in the CD4^+^ compartments of the peripheral lymphoid organs (Fig. 4A and B). It was reported that the regulatory T cells (Tregs) were increased in aged mice and altered in autoimmune disease [31, 32]. We found similar Foxp3^+^CD25^-^ and Foxp3^+^CD25^+^ regulatory T cells in both groups of 6-month-old mice, while Treg cells were significantly increased in *Aire-*deficient mice later (Fig. 4C and D). As the delayed thymic involution in the early middle-aged *Aire-*deficient mice, the elevation of Tregs should not be the inducer of thymic involution.

### Full manifestation of autoimmunity in aged Aire-deficient mice was accompanied with accelerated thymic atrophy

**Figure 5.**
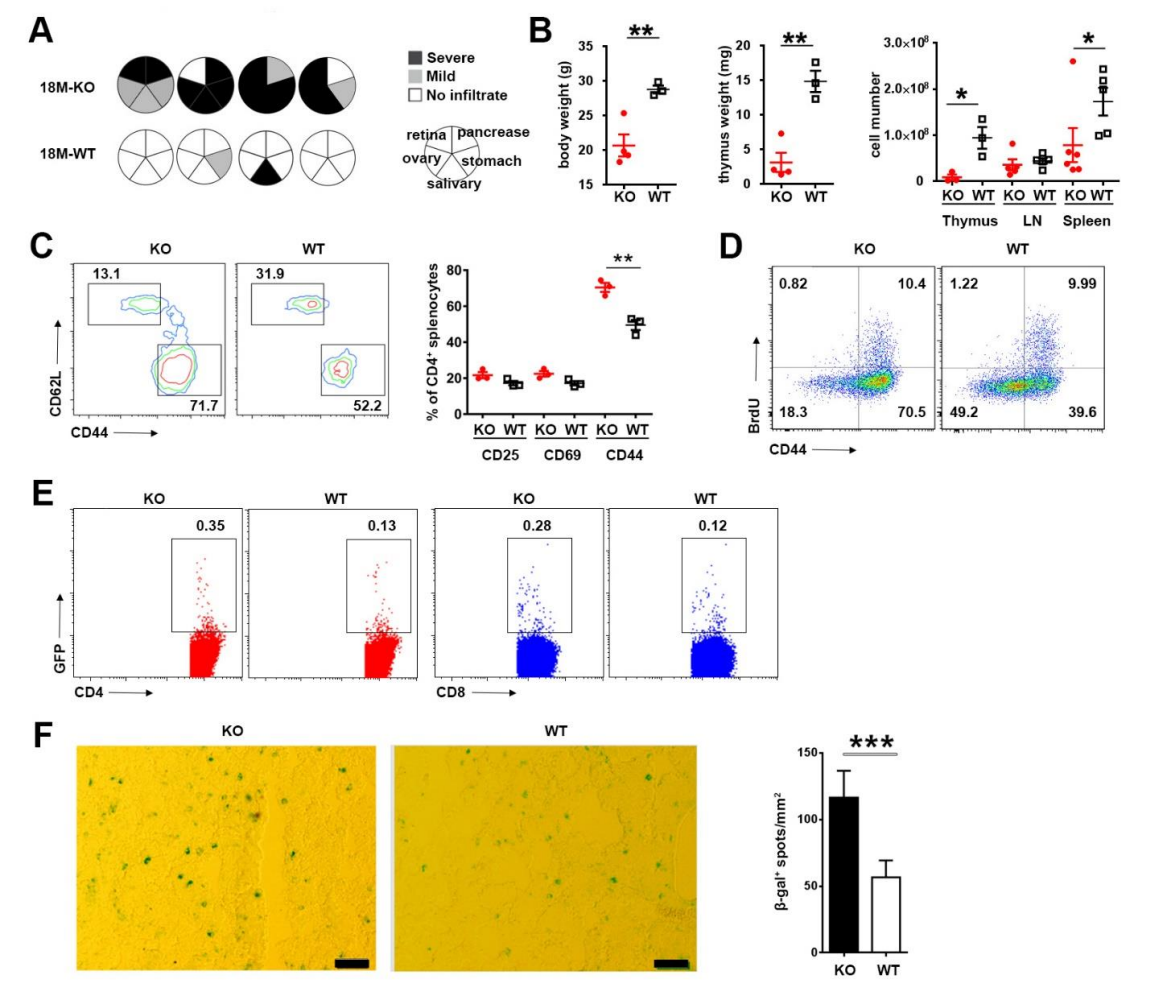
Deteriorated autoimmune disorder in aged *Aire*-deficient mice (**A**) Lymphocyte infiltration in the salivary gland, retina, pancreas, stomach and ovary in 18-month-old WT (n=4) and *Aire*^-/-^ (n=4) mice. The circles represent individual mice and summarize the degree and pattern of immune infiltration in the indicated organs. (**B**) Mouse body weight (*left*), thymus weight (*middle*) and the cell number of thymocytes, lymph node cells and splenocytes (*right*) of 18-month-old *Aire*^-/-^ mice and WT littermates. Data are presented as Mean ± SD. Each dot represents an individual mouse. (**C**) Flow cytometric analysis of T cell activated markers in the spleen of 18-month-old mice. CD62L and CD44 profile (*left*) and the percentage of CD25^+^, CD69^+^ and CD44^+^ T cells (*right*) of CD4^+^ splenocytes are shown. Data are presented as Mean ± SD (n=3 pairs). (**D**) BrdU incorporation (gated on CD4^+^ cells) in the spleen of 18-month-old mice was detected by flow cytometry. The number indicates the percentage of cells in each quadrant. (**E**) The percentage of GFP^+^ RTEs in splenic CD4^+^ and CD8^+^ T cells in 18-month-old mice. (**F**) β-gal was stained in thymic frozen slices from 18-month-old mice (*left*). Scale bars: 50μm. The number of β-gal^+^ senescent cells (blue) in 10 randomly chosen sections were counted using Image J. The statistical data are shown as average β-gal^+^ spots/mm^2^±SD (*right*, n=5 pairs). The experiments were repeated at least three times. Statistical differences between groups were determined by the Student’s *t* test. *p < 0.05, **p < 0.01, ***p < 0.001.

As the mice aged, the autoimmune symptom induced by *Aire* deficiency was progressively aggravated. In comparison to the relatively mild autoimmune signs in young and middle-aged mice (Fig. 2A and B), much more severe lymphocyte infiltration was detected in multiple organs in *Aire*^-/-^ mice at the age of 18 months and beyond (Fig. 5A), which was accompanied by a significant decrease in body weight (Fig. 5B). Furthermore, we analyzed the activation status of peripheral CD4^+^ T cells. An increased proportion of CD44^hi^ cells was found in aged *Aire* KO mice, whereas the proliferative capacity of these cells remained unaltered (Fig. 5C and D).

Notably, while the thymic weight and thymocyte number were comparable in young and middle-aged *Aire*^-/-^ and WT mice (Fig. 2C and D), aged *Aire*^-/-^ mice showed markedly reduced thymic weight and thymocyte number in comparison to age-matched WT mice (Fig. 5B). As much as thymic output was concerned, we observed even opposite effects at different ages. While a higher percentage of GFP^+^ RTEs was present in *Aire*^-/-^ than WT mice at the age of 6 or 12 months (Fig. 3A and B), fewer thymocytes were exported from *Aire*^-/-^ than WT thymus at the age of 18 months (Fig. 5E). The opposite trend of change was also seen in β-gal staining. In contrast to the reduction of β-gal^+^ cells in young *Aire-*deficient thymi (Fig. 1, 2I and J), such cells were found to be more abundant in *Aire*^-/-^ than WT mice at advanced ages (Fig. 5F). Together, these results suggest that the *Aire*^-/-^ thymus underwent accelerated degeneration once the autoimmune phenotype was fully developed.

### Increased thymic homing of IFN-γ-producing activated T cells in aged Aire-deficient mice

**Figure 6.**
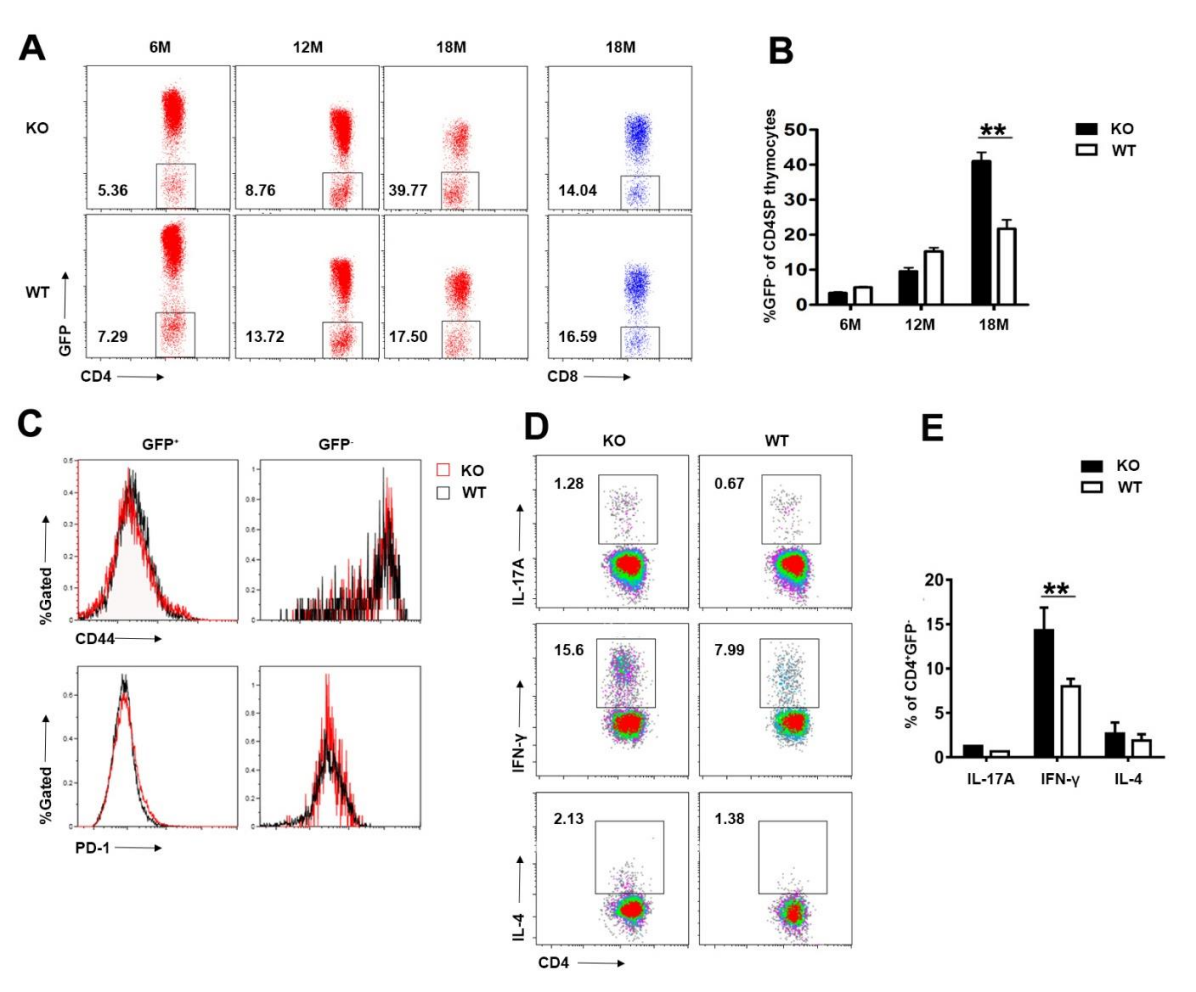
Elevated returning of IFN-γ-producing cells in aged *Aire*-deficient mice (**A, B**) Flow cytometric analysis of GFP^-^ returning T cells in the thymus of 6-, 12- and 18-month-old *Aire^-/-^* Rag2p-GFP mice and WT littermates. Representative dot plots (**A**) and the proportion of GFP^-^ T cells in CD4^+^ and CD8^+^ T cells in the thymus (**B**) are shown. (**C**) The expression levels of CD44 and PD-1 measured by flow cytometry in GFP^+^ CD4 SP thymocytes and GFP^-^ returning CD4^+^ T cells from18-month-old *Aire*^-/-^ mice and WT littermates. Representative histograms are shown. (**D, E**) Isolated CD4^+^GFP^-^ returning T cells were stimulated with anti-CD3 and anti-CD28 for 12 hours. IFN-γ-, IL-4- and IL-17A-producing cells in CD4^+^GFP^-^ returning T cells were analyzed by flow cytometry. Representative density plots (**D**) and the proportion of IFN-γ-, IL-4- and IL-17A-producing cells in CD4^+^GFP^-^ returning T cells (**E**) are shown. The experiments were repeated three times. Data are presented as Mean ± SD (n=8 pairs). Statistical differences between groups were determined by the Student’s *t* test. **p < 0.01.

It has long been known that activated T cells are able to recirculate into the thymus from the periphery and their representation increases with age [33]. We have previously demonstrated that thymic homing of activated CD4^+^ T cells has a deteriorating effect on development and maintenance of the thymic epithelium [34]. We speculated that similar mechanisms could contribute to the accelerated thymic senescence in aged *Aire*^-/-^ mice. *Aire^-/-^* Rag2p-GFP mice were used to distinguish GFP^+^ developing thymocytes from GFP^-^ recirculating T cells. As shown in Figure 6A and B, while similar percentages of GFP^-^ cells were detected in the thymic CD4^+^ population in *Aire*^-/-^ mice and the WT littermates at the age of 6 and 12 months, a much-increased representation of CD4^+^GFP^-^ thymic returning T cells were seen in 18-month-old knockout mice. When analyzed for CD44 and PD-1 expression, CD4^+^GFP^-^ cells showed higher expression of both CD44 and PD-1. No difference, however, was observed between the *Aire^-/-^* and WT groups (Fig. 6C). These data indicate increased homing of activated CD4^+^ T cells in aged *Aire-*deficient mice.

Next, we sought to determine what was the dominant subset of Th cells that homed to thymus. To this end, thymic CD4^+^GFP^-^ cells were purified and assessed for production of signature cytokines following *in vitro* stimulation with anti-CD3 and anti-CD28 for 12 hours. The majority of these cells produced IFN-γ both in *Aire*^-/-^ and WT mice, indicating the dominance of Th1 cells among thymic recirculating T cells. Notably, *Aire-*deficient mice contained a significantly higher percentage of IFN-γ-producing Th1 cells. This is consistent with the previous finding that APS1 is predominantly a Th1-mediated disorder [35].

### Induction of thymic epithelial cell senescence by Th1-biased condition

**Figure 7.**
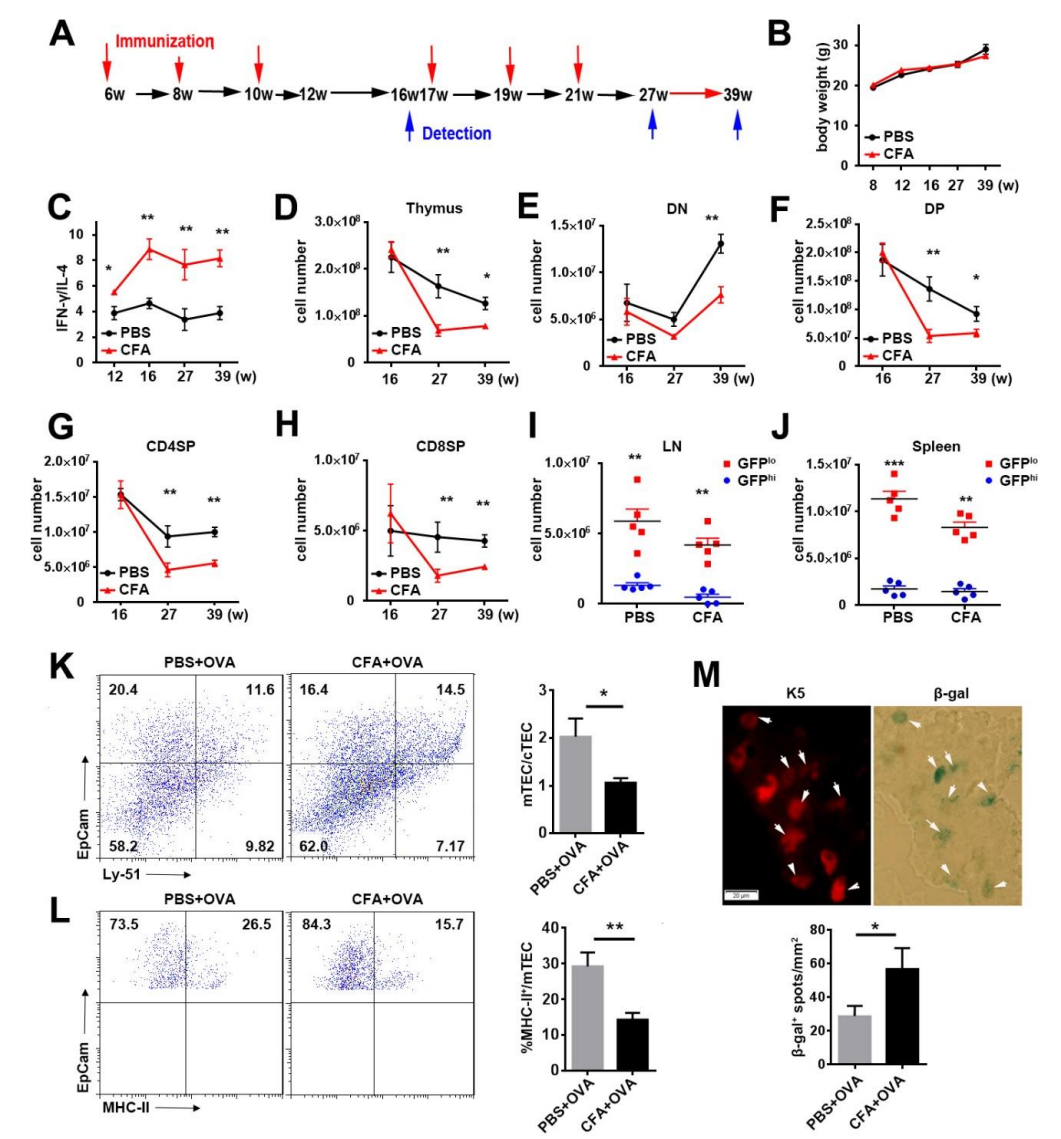
Th1 biased condition accelerates senescence of thymic epithelial cells (**A**) Mice were immunized subcutaneously with 5μg of OVA in saline or CFA for a total 6 times at 6-, 8-, 10-, 17-, 19- and 21-week-old and analyzed at 16-, 27- and 39-week-old. (**B**) Mouse body weight of immunized mice at indicated time points. (**C**) IFN-γ- and IL-4-producing CD4^+^ cells in the spleen of immunized mice at indicated time points were detected by intracellular staining. Data are showed as the ratio of IFN-γ^+^%:IL-4^+^%. (**D-H**) The numbers of total thymocytes (**D**), DN (**E**), DP (**F**), CD4SP (**G**) and CD8SP (**H**) thymocytes in the thymus are shown. (**I, J**) The percentage of GFP^+hi^ and GFP^-lo^ cells in the lymph nodes (**I**) and spleen (**J**) after OVA immunization. Each dot represents an individual mouse. (**K**) EpCam^+^Ly51^lo^ mTEC and EpCam^+^Ly51^hi^ cTEC in the thymus of two immunized groups were detected by EpCam and Ly51 staining in CD45^-^ gate. Representative density plots (*left*) and ratio of mTECs versus cTECs (*right*) are shown. (**L**) The expression of MHCII in mTEC from two immunized groups. Representative density plots (*left*) and the proportion of MHCII^+^ mTECs (*right*) are shown. (**M**) K5 (*upper left*) and β-gal (*upper right*) was stained in thymic frozen slices from CFA group. Representative images (*up*) and the percentage of β-gal^+^ cells (*down*) are shown. The white arrows pointed cells coexpressed K5 and β-gal. Scale bars: 20μm. The experiments were conducted twice with 5 mice for each group. Data are presented as Mean ± SD. Statistical differences between groups were determined by the Student’s *t* test. *p<0.05, **p<0.01, ***p < 0.001.

IFN-γ induced by experimental *Trypanosoma cruzi* infection inhibits thymic cell proliferation and promotes thymic atrophy [36]. To examine whether Th1-type thymic recirculating T cells contribute to thymic epithelial cell senescence in aged mice in vivo, we used an immunization model with complete Freund’s adjuvant (CFA). CFA is a potent adjuvant to generate a Th1-biased response [20]. C57BL/6 or Rag2p-GFP mice at 6-week of age were sequentially immunized with ovalbumin (OVA) in PBS or CFA at an interval of 2 weeks (Fig. 7A). The body weight was comparable between the two immunization groups (Fig. 7B). After 3 rounds of immunization, T cells in CFA but not PBS group showed a stable Th1-biased cytokine profile as evaluated by IFN-γ/IL-4 ratio (Fig. 7C). We thus compared the number of thymocytes in OVA/PBS- and OVA/CFA-receiving mice six weeks after 3 or 6 rounds of immunization. After the third immunization at the age of 16 weeks, no difference was found between CFA and PBS groups (Fig. 7D-H). After the sixth immunization at the age of 27- and 39-weeks, we detected a significant decrease in the number of total thymocytes, double negative (DN), DP, CD4 single positive (SP), and CD8SP thymocytes in CFA group (Fig. 7D-H). Consistent with the reduction of thymocytes, decreased numbers of RTEs in the lymph nodes and spleen were also observed in CFA group (Fig. 7I and J). The proportion of thymic returning GFP^-^CD4^+^ T cells was increased at 6 weeks after the final immunization. Most of these returning T cells were IFN-γ-producing cells (data not shown).

The reduced MHC-II expression is a hallmark of thymic senescence [37]. Compared to the mice immunized with OVA in PBS, the ones with OVA in CFA showed significantly reduced ratio of EpCam^+^Ly51^lo^ mTECs versus EpCam^+^Ly51^hi^ cortical thymic epithelial cells (cTECs) and decreased MHC-II^+^ cells in mTECs (Fig. 7K and L), indicating a decline of mTEC numbers and functions. In addition, the β-gal^+^ cells, majority of which were K5^+^ epithelial cells, were significantly increased in thymic slices obtained from CFA group (Fig. 7M). These data further demonstrate that mTEC senescence can be induced by thymic homing of IFN-γ-producing CD4^+^ cells

## DISCUSSION

Thymic aging results in less efficient T cell development and decreased thymic output [1] and is linked to increased morbidity and mortality in a wide range of clinical settings such as opportunistic infections, autoimmunity and the incidence of cancer. Whether thymic degeneration is causative for or a response in the pathologic state is still an open question and an active area of investigation. We demonstrated that thymic involution did not occur around the onset of autoimmune diseases in middle-aged *Aire*-deficient mice. Th1-biased peripheral environment for long duration of time and increased thymic returning IFN-γ-producing CD4^+^ T cells in aged *Aire-*deficient mice accelerated TEC senescence and thymic involution.

Premature aging of the thymus is one of the major immunological phenotypes in young patients with autoimmune diseases such as RA, SLE, and systemic sclerosis [9, 12, 13, 38], indicating that chronic inflammation may accelerate thymic involution. Th1 cells play a critical role in autoimmune pathogenesis. Increased IFN-γ mRNA could be detected in the thymus tissue of aged myasthenia gravis patients [39]. We also found IFN-γ-producing peripheral and thymic returning Th1 cells in *Aire-*deficient mice or mice immunized with antigen in CFA. The accumulation of these Th1 cells in the thymus was closely associated with reduced thymocyte numbers and thymic output and increased senescent epithelial cells in these mouse models. Consistently, patients with autoimmune polyendocrinopathy-candidiasis-ectodermal dystrophy (APECED, a human form of *Aire* mutation) also have Th1-biased cytokine profile [35], suggesting that Th1 cells contribute to accelerated thymic aging in this specific autoimmune disorder. In addition, the impact of IFN-γ on senescence is not limited to thymic epithelial cells. It was found that IFN-γ could induce senescence-like characteristics in mouse bone marrow mesenchymal stem cells [40]. The accumulation of IFN-γ-producing effector CD4^+^ T cells in the mediastinal lymph nodes contribute to myocardial aging [41]. Together, these data suggest that the progressively increased peripheral Th1 cells in the patients with autoimmune diseases facilitate their reentry into the thymus and contribute to accelerated thymic involution.

Both IL-17 and IL-6 contribute to arthritis development at the early onset of RA [42, 43]. Thymic epithelial cells can also produce several IL-6 family cytokines, particularly in aged normal human thymus. When exogenously administered to young mice, these cytokines induced rapid and acute thymus gland involution and decreased thymic export to the periphery [39]. However, comparable levels of IL-6 family cytokines including *Il6*, leukemia inhibitory factor (*Lif*), and oncostatin M (*Osm*) were found in WT and *Aire-*deficient thymic epithelial cells (Supplemental Fig. 1), suggesting that these cytokines may not contribute to premature thymic involution at least in *Aire*-deficient mice.

Notably, the autoimmune pathology is very mild in 6-month-old *Aire*-deficient mice on C57BL/6 background [44], even though the susceptibility to EAE is increased when compared to 2-month-old mice [45]. The decrease in thymocyte numbers and output did not occur until the mice reach 18 months of age and had severe inflammatory cell infiltration. It suggests that thymic involution occurs later than the onset of autoimmune disease, likely in waiting for Th1 cells to be accumulated in the thymus [46].

Taken together, our finding demonstrates an important link between pathogenic mechanism of autoimmune disease and premature thymic involution. It further suggests that early intervention in young autoimmune disease patients with Th1-biased profile may delay the onset of the disease by postponing the thymic involution.

## Supplemetary Material

The Supplemenatry material for this article can be found online at: www.aginganddisease.org/EN/10.14336/AD.2018.0608


