# Supplementary material for: Th1 Biased Progressive Autoimmunity in Aged Aire-Deficient Mice Accelerated Thymic Epithelial Cell Senescence
Source: Aging Dis. 2019 Jun 1;10(3):497–509. doi: 10.14336/AD.2018.0608 (PMC6538216; doi:10.14336/AD.2018.0608)
Supplement: Supplementary file 1 [file AD-10-3-497-s.pdf]

SUPPLEMENTARY DATA

Th1 Biased Progressive Autoimmunity in Aged *Aire*-Deficient Mice Accelerated Thymic Epithelial Cell Senescence

Jie Zhang, Yuqing Wang, Abudureyimujiang Aili, Xiuyuan Sun, Xuewen Pang, Qing Ge, Yu Zhang*, Rong Jin*

Department of Immunology, School of Basic Medical Sciences, Peking University Health Science Center, Key Laboratory of Medical Immunology, Ministry of Health (Peking University), Beijing, 100191, China.

© 2018. Zhang J et al. Published online at http://www.aginganddisease.org/EN/10.14336/AD.2018.0608

SUPPLEMENTARY DATA

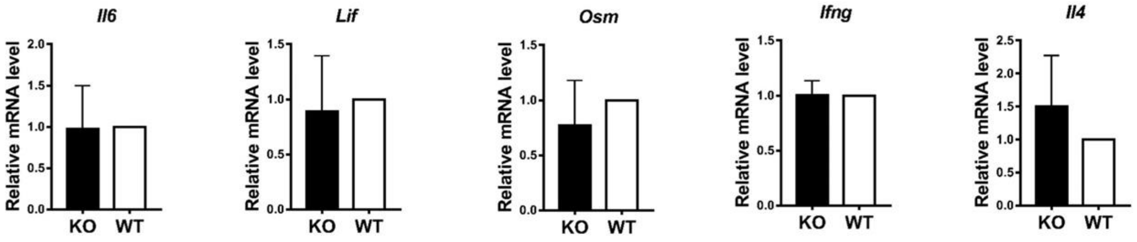

Supplementary Figure 1. The cytokines production in *Aire*-deficient mice at early-middle aged stage. The mRNA levels of *Il6*, *Lif*, *Osm*, *Ifng* and *Il4* in TECs of 6-month-old *Aire*-deficient mice and WT littermates were determined by quantitative PCR. The experiments were repeated 3 times with duplicates for each sample. Data are presented as Mean ± SEM. Statistical differences between groups were determined by the Student's *t* test. No significance was detected in all compared groups.

© 2018. Zhang J et al. Published online at http://www.aginganddisease.org/EN/10.14336/AD.2018.0608
